# *MTHFR* C677T and A1298C Polymorphisms in Breast Cancer, Gliomas and Gastric Cancer: A Review

**DOI:** 10.3390/genes12040587

**Published:** 2021-04-17

**Authors:** Igor Petrone, Paula Sabbo Bernardo, Everton Cruz dos Santos, Eliana Abdelhay

**Affiliations:** 1 Stem Cell Laboratory, Center for Bone Marrow Transplants, Brazilian National Cancer Institute—INCA, Rio de Janeiro 20230-240, Brazil; evertoncruzsantos@gmail.com (E.C.d.S.); eabdelhay@inca.gov.br (E.A.); 2 Stricto Sensu Graduate Program in Oncology, INCA, Rio de Janeiro 20230-240, Brazil; paulasabbo@gmail.com; 3 Laboratory of Cellular and Molecular Hemato-Oncology, Molecular Hemato-Oncology Program, Brazilian National Cancer Institute—INCA, Rio de Janeiro 20230-240, Brazil

**Keywords:** polymorphism, *MTHFR*, breast cancer, glioma, gastric cancer

## Abstract

Folate (vitamin B9) is found in some water-soluble foods or as a synthetic form of folic acid and is involved in many essential biochemical processes. Dietary folate is converted into tetrahydrofolate, a vital methyl donor for most methylation reactions, including DNA methylation. 5,10-methylene tetrahydrofolate reductase (MTHFR) is a critical enzyme in the folate metabolism pathway that converts 5,10-methylenetetrahydrofolate into 5-methyltetrahydrofolate, which produces a methyl donor for the remethylation of homocysteine to methionine. *MTHFR* polymorphisms result in reduced enzyme activity and altered levels of DNA methylation and synthesis. *MTHFR* polymorphisms have been linked to increased risks of several pathologies, including cancer. Breast cancer, gliomas and gastric cancer are highly heterogeneous and aggressive diseases associated with high mortality rates. The impact of *MTHFR* polymorphisms on these tumors remains controversial in the literature. This review discusses the relationship between the *MTHFR* C677T and A1298C polymorphisms and the increased risk of breast cancer, gliomas, and gastric cancer. Additionally, we highlight the relevance of ethnic and dietary aspects of population-based studies and histological stratification of highly heterogeneous tumors. Finally, this review discusses these aspects as potential factors responsible for the controversial literature concerning *MTHFR* polymorphisms.

## 1. Introduction

Folate is a water-soluble vitamin (vitamin B9) found in some foods (e.g., okra, broccoli, asparagus, lentils, and spinach) or in a synthetic form (folic acid) used in fortification and dietary supplementation programs [1,2]. This vitamin is a limiting factor in the methylation reactions of DNA, RNA and proteins. Plants and bacteria can synthesize folic acid, but not humans and other animals; the latter must synthesize folic acid through the diet. The hydrophilic and anionic characteristics of folate at physiological pH prevent its passive diffusion through the plasma membrane. At least three types of intracellular folate carrier proteins have been identified: a specific anion transporter to the reduced form with ubiquitous tissue distribution; a glycosyl-phosphatidylinositol (GPI) carrier bound to high-affinity proton in an acidic pH environment and a transporter of high-affinity GPI anchored proteins [3].

Folates in nature are present in a reduced form. The in vivo biological activation of folic acid requires a reduction in the intermediate forms of dihydrofolate and tetrahydrofolate by adding hydrogen atoms in the pyrazine ring and pteridine at positions 7, 8 and 5, 6, 7 and 8, respectively. Folate can also bind to the carbon units of methyl groups (CH3), methylene (CH2), formyl (–CHO–) or formimino (–CHNH–) at positions N5, N10, or both positions of the structure. This process imparts function to folate coenzyme in various enzymatic systems as a carbon carrier unit at different degrees of oxidation. Carbon donor unit function is essential for methylation reactions, nucleotide synthesis, DNA synthesis and repair [4,5,6] and can influence carcinogenesis by adverse effects on global DNA methylation and/or promoter regions of specific CpG sites [7]. In addition to the effects of methylation in DNA and possible subsequent damage to molecules, disturbances in the metabolism of carbon-1 cause DNA damage via effects on nucleotide synthesis. In folate deficiency, uracil is incorporated into DNA molecules; during repair, breaks may occur in DNA molecules by uracil glycosylase (UDG), causing damage and chromosomal translocations, a hallmark of genomic instability that may contribute to tumor progression [7,8,9].

**Figure 1:**
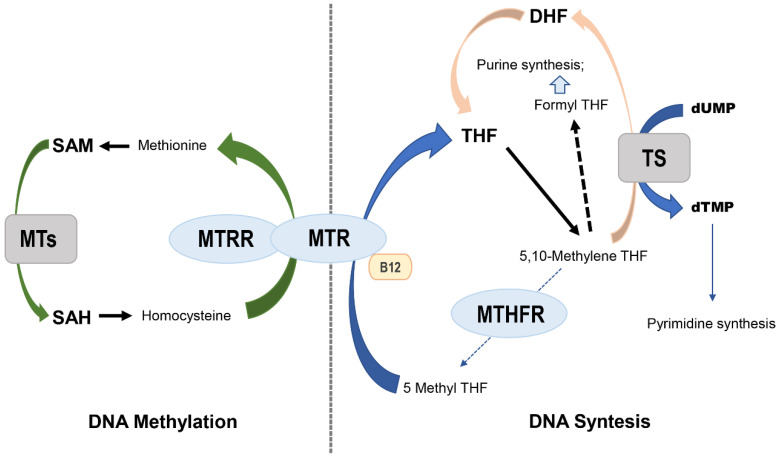
Schematic of folate metabolism. Effects of reduced MTHFR activity on DNA synthesis and methylation. DHF, dihydrofolate; MTs, methyltransferases; THF, tetrahydrofolate; SAM, S-adenosyl methionine; SAH, S-adenosyl homocysteine; TS, thymidylate synthase. MTR, methionine synthase; MTRR, methionine synthase reductase.

MTHFR (5,10-methylene tetrahydrofolate reductase), MTR (methionine synthase) and MTRR (methionine synthase reductase) are key enzymes in the metabolism of folate. MTHFR occupies a central position while maintaining homeostasis between DNA synthesis and methylation (Figure 1), promoting irreversible conversion of 5,10 methylene tetrahydrofolate to 5-methyltetrahydrofolate [10]. The substrate 5,10 methylene tetrahydrofolate is used by thymidylate synthase to methylate dUMP to dTMP. The latter is the only required source of thymidine for DNA synthesis and repair, and the product functions as a methyl donor required for the remethylation of homocysteine to methionine. This reaction is catalyzed by the MTR enzyme and dependent on vitamin B12, which acts as a carrier of intermediate methyl groups. The oxidation of vitamin B12 acts as a cofactor that may inactivate MTR, which can again be functionally active when the reductive remethylation of vitamin B12 by MTRR occurs. The reduced activity of the MTHFR pathway inhibits the production of 5-methyltetrahydrofolate and can lead to accumulation of the substrate. Decreased levels of the MTHFR product cause increased levels of homocysteine, decreased levels of blood folate and displacement means for folate synthesis and DNA repair. Circulating folate is a cosubstrate for the remethylation of homocysteine to methionine, and methionine is a precursor to S-adenosylmethionine (SAM), which is the main methyl donor. Low levels of folate and/or reduced enzyme activity of the main proteins involved in folate metabolism may result in limiting the substrate for methionine synthase, thus affecting the remethylation pathway and resulting in a high concentration of homocysteine in the plasma; high plasma levels of homocysteine have been linked to several types of human cancers [11,12,13,14,15,16,17].

DNA methylation causes the covalent modification of genomic DNA that alters gene expression and the transmission of epigenetic information that is perpetuated through DNA replication and cell division. Changes in the patterns of DNA methylation are critical features in the process of carcinogenesis, and global DNA hypomethylation and hypermethylation of specific promoters may occur. Global DNA hypomethylation is associated with low folate intake in animals and experimental studies with human models [18,19]. Aberrant changes in DNA methylation are involved in the generation and progression of a neoplastic phenotype. Three processes characterize the contribution of changes in the patterns of DNA methylation in the initiation and progression of cancer: (i) global hypomethylation promotes chromosomal instability by the reactivation of transposable elements and loss of imprinting; (ii) local hypomethylation induces the activation of oncogenes; and (iii) most of the time, hypermethylation promotes the silencing of tumor suppressor genes [19,20,21].

Breast tumors are characterized by a high incidence among women and are one of the main causes of mortality. Gliomas are heterogeneous tumors and have high rates of recurrence and mortality. Gastric cancer is the fifth most common type of tumor with the third highest mortality rate. Literature reports have demonstrated the impact of folate metabolism on DNA synthesis and methylation, resulting in an increased risk of several cancer types when this pathway is dysregulated. In this context, this review discusses the relationship of changes in the folate pathway resulting from the C677T and A1298C polymorphisms of the *MTHFR* gene with the process of breast, glioma and gastric carcinogenesis.

## 2. MTHFR

MTHFR is a key enzyme in the folate metabolism pathway that converts 5,10-methylenetetrahydrofolate to 5-methyltetrahydrofolate, the primary circulatory form of folate, producing a methyl donor for the remethylation of homocysteine to methionine in a conversion catalyzed by MTR using vitamin B12 (Figure 1) as a cofactor. Defects in the processing of folate due to decreased enzyme activity have been associated with reduced levels of circulating folate and may culminate in changes in the levels of DNA methylation and gene regulation. The occurrence of polymorphisms in enzymes encoding genes of the folate pathway provides a functional impact on metabolism [16,17,22,23,24].

The *MTHFR* gene is located at the end of the short arm of chromosome 1 (1p36.3), and the DNA sequence for this gene is approximately 2.2 kilobases (kb) and includes 11 exons [24,25]. *MTHFR* polymorphisms have been extensively investigated to assess their association in various medical conditions, primarily cardiovascular disease, thrombosis, pregnancy complications, neural tube defects, cancer risk, and psychiatric disease [16,26]. There are two polymorphisms of the *MTHFR* gene already well described as associated with numerous pathologies: C677T and A1298C [15,27,28].

## 3. *MTHFR* C677T and A1298C Polymorphisms and Cancer

The C677T polymorphism is an exchange at position 677 of the *MTHFR* gene whose exchange of nucleotide cysteine for thymine culminates in the replacement of alanine by valine in the MTHFR enzyme. This mutation is associated with reduced enzyme activity and increased thermolability of the enzyme already reported in vitro, leading to a decrease in 5-methyltetrahydrofolate and an increase in the accumulation of the substrate 5,10-methylenetetrahydrofolate. The reduction in enzyme activity leads to an increase in plasma homocysteine levels in mutant homozygous subjects and high plasma homocysteine levels; in heterozygotes, homocysteine levels are high compared with normal individuals but lower than homozygotes [28,29,30,31].

The *MTHFR* A1298C polymorphism occurs in exon 7 and results in glutamate transformation into alanine at codon 429. This polymorphism is located in the regulatory domain S-adenosyl methionine (SAM) of the enzyme and causes conformational changes in the MTHFR enzyme that also reflect a reduction in enzyme activity but do not result in a thermolabile protein [15,27]. Unlike the C677T polymorphism, the effect of the A1298C polymorphism on plasma homocysteine levels is inconsistent. However, under conditions of extreme folate depletion, this polymorphism becomes clinically relevant [32,33,34]. Many studies have investigated the association of the *MTHFR* polymorphism and pathologies. As already reported in the literature, it has been associated with vascular diseases, diabetes, neurological diseases, recurrent pregnancy loss and infertility, and psoriasis [35,36,37,38,39,40,41,42,43,44].

Studies have shown that folate deficiencies and the presence of polymorphisms in genes related to the folate pathway increase the incidence of cancer. MTHFR is directly involved in folate metabolism. Therefore, *MTHFR* polymorphisms may directly affect the incidence of cancer, likely by causing an imbalance in maintaining the epigenome. However, data regarding the association between the *MTHFR* polymorphism status and cancer risk have also been conflicting [26,45]. Several types of tumors have been associated with the presence of polymorphisms in *MTHFR*. The *MTHFR* gene polymorphisms show a relationship with leukemias and lymphomas with a more marked presence of the C677T polymorphism than A1298G in studies with significant results obtained [46,47,48,49,50].

In a meta-analysis of 134 case-control studies, the *MTHFR* C677T polymorphism was significantly associated with an increased risk of tumors, and stratified analyses showed an increased risk of stomach and esophageal cancer in addition to an increased risk in Asian ethnicities [51]. Other meta-analyses have shown an association of esophageal cancer in individuals with the *MTHFR* C677T CT and TT genotypes with low folate intake, and these effects could be largely modified using tobacco. Individuals with the *MTHFR* 1298 CC polymorphism have no significantly increased risk of esophageal cancer [52,53]. The status of the C677T homozygous polymorphism has also been linked to an increase in gastric cancer [54], and this risk is increased in individuals with low folate intake. Additional factors, such as diet, smoking and family history, may contribute to the risk.

In adenomatous polyposis, a precursor stage of colorectal adenocarcinoma, the 677TT *MTHFR* variant is associated with a reduced risk of developing carcinoma in the presence of high doses of folic acid and vitamins B6, B12 and/or B2 in subjects with a low intake of alcohol. However, the risk increases in deficient vitamin nutrition [55]. This reduces 677TT variant MTHFR enzyme activity and can deflect methyl groups available via DNA methylation in DNA synthesis, irreversibly committing 1-carbon units for the methylation cycle and away from the synthesis of thymidylate and purine [10,20]. The *MTHFR* 677TT genotype is associated with global DNA hypomethylation, which can be enhanced in situations of folate depletion. The overall hypomethylation is associated with hypermethylation of tumor suppressor genes, particularly in subjects with *MTHFR* 677TT [56,57]. The modulation of risk-associated with *MTHFR* 677TT cancer can be due to the separation of carbon from methylation cycle group-1 and synthesis of thymidine [58]. Therefore, functional polymorphisms in *MTHFR* seem to be somewhat related to several cancer types.

## 4. *MTHFR* C677T and A1298C Polymorphisms and Breast Cancer

Breast cancer (BC) is the most common cancer type among women worldwide and has a high mortality rate. It is characterized by the expression of aberrant genes that confer heterogeneous morphology and aggressiveness to the tumor in addition to varied clinical manifestations [59,60]. Genomic studies have increased the knowledge about the heterogeneity of BC, allowing its classification into four intrinsic subtypes of invasive tumors (IBCs) based on receptor expression: luminal A, characterized by the expression of estrogen and/or progesterone receptors (ER/PR); luminal HER, characterized by the expression of ER and/or PR and human epidermal growth factor receptor 2 (HER-2); HER-2, characterized by HER-2 overexpression and the absence of ER and PR; and the triple-negative (TN) subtype, which does not express any of these three receptors [61,62]. Ductal carcinoma in situ (DCIS) is a noninvasive BC type that can evolve to invasive types, has been molecular characterized similarly and has been described as a possible invasive precursor of breast tumorigenesis, although this classification and the potential to become invasive remain controversial in the literature [63].

Several environmental and genetic elements are involved in different BC types, and various mutations in tumor suppressor genes and oncogenes are involved in the process of carcinogenesis [64]. Thus, polymorphisms may be responsible for the susceptibility to BC among different populations, and studying them is critical to better understand the biology of the tumor, to develop new strategies for the diagnosis or seek more effective therapies for treatment. Importantly, variations in the *MTHFR* genotype reduce the catalytic activity of MTHFR, and this activity is essential for DNA synthesis, methylation and repair [65]. Functional polymorphisms in *MTHFR* have been shown to influence the risk of BC, although this finding remains controversial, and the data from the evaluated studies are shown in Table 1.

In a case-control study conducted in China with 560 patients diagnosed with BC and 560 healthy individuals, a significant association was observed between the homozygous TT genotype for the C677T polymorphism of the *MTHFR* gene and the risk of BC compared with the wild homozygous genotype CC (*p* = 0.007). Additionally, the CC genotype reduced the risk of the breast carcinogenesis process (*p* = 0.004) in addition to reducing the risk of death compared with the TT genotype [66]. Another group showed a 1.7 times higher incidence of breast cancer in individuals with the polymorphic T allele and 2.5 times higher for homozygous TT individuals than for individuals with the wild genotype. However, they were not observed in the distribution of the A1298C *MTHFR* polymorphism between cases and controls in the risk of BC [67].

Analyses of the C677T polymorphism from peripheral blood samples obtained from 100 Iranian women with BC and 142 healthy women showed that the T allele and TT genotype had a higher prevalence in patients (*p* < 0.0001). Furthermore, the polymorphic genotype correlated strongly with the risk of BC (*p* < 0.0001) and correlated with women in the menopausal period (*p* = 0.036), although with no relationship to age, weight, body mass or height. These results corroborate previous data from Gao et al., who showed an association between polymorphic homozygotes and the risk of BC [68,69]. Sohn et al. previously verified the role of the single-nucleotide polymorphism (SNP) T of the C677T polymorphism in vitro in folate metabolism as a risk factor for BC and suggested the use of SNPs as possible pharmacogenetics for chemotherapy treatment [70]. Controversially, a study of 1459 women aged between 25 and 64 years revealed no statistically significant correlation between the risk of BC and the C677T polymorphism, although breast cancer was more prevalent in women with the T allele and low folate intake, suggesting that the polymorphic allele can be considered an important risk factor for breast cancer [71].

A meta-analysis publication performed by He *and colleagues* observed a strong tendency toward the risk of BC in women with polymorphic homozygous (TT) and heterozygous (CT) genotypes for the *MTHFR* C677T polymorphism. This risk was increased in women of Asian and Caucasian ethnicities, suggesting that polymorphism may be a risk factor for tumorigenesis [72]. However, another publication that evaluated a pooled control and meta-analysis case study observed no association between the replacement of cytosine by thymine at position 677 of the *MTHFR* gene, although the risk of BC in the presence of the polymorphism was higher among Caucasian and Asian populations of the evaluated groups [73]. Analyses of another meta-analysis study that evaluated 57 other studies suggested a positive association between the C677T polymorphism and the risk of BC, particularly in Asian populations (odds ratio = 0.942). However, this meta-analysis provided evidence that the A1298C polymorphism would not be significantly associated with BC risk [74].

Considering the possible relationship between the polymorphism and Caucasian and Asian ethnicities, another group evaluated the influence of the C677T and A1298C polymorphisms in Latin American populations and found a high risk for the development of BC in homozygous, recessive and allelic genetic models for the C677T polymorphism (*p* = 0.02, *p* = 0.04, and *p* = 0.01, respectively). However, no association was observed between the A1298C polymorphism and the risk of developing BC in Latino American populations [75]. In a study performed using DNA extracted from the peripheral blood of 100 Brazilian women with BC and 144 healthy women, an association between the recessive model of the C677T polymorphism (TT) and an increased risk of BC was observed (*p* = 0.03). These data corroborated previous case-control studies of Chinese women who also showed a risk of developing BC in women with the 677TT genotype [76,77,78]. Analyses of the CT + TT genotypes together versus TT in Moroccan and Kazakhstan women suggest a relationship of only one polymorphic allele with the increased risk of BC, and this influence of the allele was not observed in Brazilian women [76,79,80]. Interestingly, the AC and CC genotypes of the A1298C polymorphism have been linked to a decreased risk of BC in Kazakhstan women [79]. However, the association of the 677TT polymorphism with alcohol consumption and BC incidence was observed in Brazilian women aged older than 50 years. By contrast, the relationship between the increased risk for BC was not observed in women with the homozygous polymorphic genotype TT and body mass index and tobacco consumption [76].

**Table 1:** Effect of the polymorphisms on breast cancer risk.

Study Type	Number of Participants	Ethnicity	SNP	Risk Association	References
Case-control	560 BC cases and 560 controls	Chinese	C677T	Significant association of the TT genotype with the risk of BC.	[66]
Case-control	150 BC cases and 150 controls	Jordanian	C677T A1298C	Influence of the T allele and TT genotype on the risk of BC. No significant differences for A1298C in BC risk.	[67]
Case-control	100 BC cases and 142 controls	Iranian	C677T	Correlation of the T allele and TT genotype with the risk of BC and menopause status.	[68]
Case-control	669 BC cases and 682 controls	Chinese	C677T A1298C	TT genotype associated with BC risk. Low folate intake associated with the susceptibility to BC at 1289A/A, 677C/C and 677C/T.	[69]
Case-control	1459 BC cases and 1556 controls	Chinese	C677T	Highest prevalence of BC was observed in women with the T allele and low folate intake.	[71]
Meta-analysis	19,260 BC cases and 23,364 controls	Asian and Caucasian	C677T	Strong trend toward the risk for BC in the TT and CT genotypes. Increased risk in Asian and Caucasian populations.	[72]
Case-control and Meta-analysis pooled	28,619 BC cases and 32,388 controls	Indian	C677T	No association between the exchange of T for C at position 677 of *MTHFR*.	[73]
Meta-analysis	25,877 BC cases and 29,781 controls	Asian, Caucasian and Mixed population	C677T A1298C	Positive association between C677T and the risk of BC, particularly in Asian populations. Non-significant association of A1298C with BC. The A allele affects BC risk in Caucasian populations and a reverse effect in Asian and mixed populations.	[74]
Meta-analysis	3362 BC cases and 4175 controls	Latin American	C677T A1298C	High risk of BC for C677T. No evidence of a relationship between the A1298C polymorphism and BC.	[75]
Case-control	100 BC cases and 144 controls	Brazilian	C677T	Significant association of TT with an increased risk of BC. Relationship between alcohol consumption and the incidence of BC.	[76]
Case-control	315 BC cases and 604 controls	Kazakhstan	C677T A1298C	CT and TT genotypes associated with an increased risk of BC. AC and CC genotypes associated with a decreased risk of BC.	[79]
Case-control	96 BC cases and 117 controls	Moroccan	C677T	T allele and TT genotype associated with increased risk for BC. Correlation between polymorphism and PR expression.	[80]
Case-control	253 BC cases and 257 controls	Brazilian	C677T A1298C	C677T associated with metastasis and ER expression. AA genotype associated with higher degrees of BC and ER expression.	[81]
Meta-analysis	19,527 BC cases and 23,123 controls	Asian, Caucasian and Mixed population	A1298C	Association between the C allele and CC genotype and increased risk for BC.	[82]
Case-control	61 BC cases and 63 controls	Brazilian	C677T A1298C TYMS	No association between C677T and A1298C polymorphisms and BC. Association between TYMS polymorphism with risk of more aggressive BC subtypes	[83]
Case-control	58 BC cases and 58 controls	Italian	C677T A1298C	Polymorphisms related to BC progression.	[84]
Case-control	610 BC cases and 1207 controls	North American	C677T A1298C	No association between polymorphisms and BC. Relationship between folate levels and BC.	[85]
Case-control	100 BC cases and 60 controls	Egyptian	C677T A1298C	Association between TC mutant haplotype and BC aggressiveness.	[86]
Case-control	124 BC cases and 63 controls	Asian	C677T A1298C	TT genotype associated with BC risk in advanced age patients	[87]

Another group that evaluated 253 Brazilian women with BC and 257 healthy women aged older than 50 years found an association of the C677T polymorphism with distant metastases (CC versus CT + TT, *p* = 0.028; CT versus CC + TT, *p* = 0.031) and with the expression of ER (*p* = 0.02). Interestingly, analyses of the A1298C polymorphism performed by the same group showed an association of the wild-type homozygous (AA) genotype with higher degrees of severity of sporadic breast cancer given the lower prevalence of patients with this genotype in stage 0 of the disease. The authors suggested that the C677T and A1298C polymorphisms were not associated with the risk of BC, but might modulate the severity of the disease [81]. Conversely, the results of another meta-analysis showed an increased risk of BC associated with the C allele of the A1298C polymorphism, particularly in Caucasians and Asians when stratifying ethnic analyses. Additionally, the polymorphic homozygote (CC) showed a higher risk of developing BC than the wild homozygote (AA), suggesting a significant association of the MTHFR A1298C polymorphism with the risk of developing BC [82]. In a recent case-control study carried out with women from the North of Brazil, no significant associations were observed between the C677T and A1298G polymorphisms with the risk of BC. However, the group found a strong association between polymorphism in a gene encoding thymidylate synthase (TYMS) and the risk of developing more aggressive BC subtypes. Importantly, TYMS displaces the accumulated MTHFR substrate (5,10-methylenetetrahydrofolate) in response to decreased enzyme activity for DNA synthesis and contributes to methylation imbalance and the results of this group suggest that the decrease in MTHFR activity and the levels of global methylation can occur in response to the displacement of the substrate for DNA synthesis in response to TYMS polymorphisms [83].

In a case-control study with 58 women with IBC and 58 healthy women, a trend was observed for the association between the most aggressive BC biotypes (HER-2 and TN) and the mutant allele variants of the C677T and A1298C polymorphisms. The association of the biophenotype and polymorphisms highlighted the risk of presenting a more aggressive biophenotype two or three times higher in patients with the C677T and A1298C polymorphisms, respectively, suggesting an association of the polymorphisms with tumor progression. Additionally, the group also showed an association of the A1298C polymorphism with the risk of lymph node metastasis and an increased risk of developing BC in patients with polymorphic homozygous (CC) genotypes and overweight individuals [84]. However, Houghton et al. did not observe a significant association between polymorphisms and BC, although relating the presence of the polymorphism to plasma folate levels has shown a strong relationship with BC. Additionally, the folate levels were significantly associated with invasive BC and positivity for hormone receptors (ER and PR) [85]. Another case-control study carried out in Egyptian women showed a strong association between CT/TT genotypes as well as AC/CC with BC susceptibility. In addition the strong presence of the TC mutant haplotype associated with overexpression of the HER-2 receptor, the presence of tumor cells in the lymph nodes and the size of the tumor suggests a relationship between the C677T and A1298G polymorphisms with BC aggressiveness and the use of the evaluation of these polymorphisms as possible markers predictive and prognostic factors for the development of BC [86]. Recently, a case-control study in a South Asian population showed for the first time an association between the homozygous polymorphic T genotype of the C677T polymorphism and the risk of breast cancer in older patients (>35 years) suggesting that the reduction of enzyme activity of MTHFR for breast cancer risk may be related, among other epigenetic factors, to age. Interestingly, in the same study, the lack of the CC genotype in patients suggests a protective effect of the A1298C polymorphism against breast cancer [87]. More robust studies that include pre-invasive breast cancer phenotypes (DCIS) are needed to better standardize the relationship of these polymorphisms with the risk of BC.

## 5. *MTHFR* C677T and A1298C Polymorphisms and Glioma

Central nervous system (CNS) tumors represent approximately 1.4% of the global tumor burden, with an increasing incidence worldwide [88,89]. The global incidence of malignant brain tumors is estimated to be 4.25–5.57 cases per 100,000 habitants, accounting for 960,000 cases per year [90,91]. Despite the low incidence, malignant brain tumors are associated with high morbidity and mortality rates [92]. Brain and other CNS tumors are highly heterogeneous; according to the World Health Organization (WHO) histological classification, they are divided into four malignancy grades [93]. In 2016, WHO published an integrated classification based on histological and molecular parameters, representing a breakthrough for CNS tumor diagnoses, mainly for gliomas [94]. Gliomas can be divided into oligodendroglioma, astrocytoma, ependymoma and mixed tumors depending on the affected glial cell type [93]. Among them, glioblastoma (GBM) is the most aggressive and malignant subtype of astrocytoma (WHO grade IV) [94]. Meningiomas and gliomas are the most common CNS tumors, representing 37.6% and 25.5% of these tumors, respectively [92]. Meningioma is the most frequent nonmalignant tumor (53.3%), while GBM is the most frequent malignant one (48.3%).

WHO classification influences treatment choices and is a predictor of tumor biological behavior. Molecular classification provides insights to understand disease initiation, progression, and the development of targeted therapies. In this context, understanding the controversial impact of genetic polymorphisms involving folate metabolism in brain tumors is relevant. As previously detailed, *MTHFR* C677T and *MTHFR* A1298C polymorphisms confer lower functioning MTHFR enzymes and are associated with increased cancer risk [95]. The production of the universal methyl donor SAM is dependent on MTHFR, which is a rate-limiting enzyme in this process. Additionally, intracellular SAM production is reduced by methyl-deficient diets and an inadequate supply of folate and vitamins B6 and B12, leading to genomic hypomethylation [96]. Several studies have associated genomic hypomethylation with tumorigenesis by promoting genomic instability, the loss of imprinting and oncogene activation [97,98]. Thus, we intend to summarize and critically explore available literature reports on *MTHFR* polymorphisms in CNS tumors. The studies evaluated in this review and their results are shown in Table 2.

In pediatric tumors, a case-control study of 73 Thai children with different types of brain tumors demonstrated that the CC allele of the *MTHFR* A1298C polymorphism was associated with a 3.9-fold increased risk of embryonic tumors such as medulloblastoma, pineoblastoma and primitive neuroectodermal tumor (PNET). This polymorphism was not associated with an increased risk of glial and germ cell tumors [99]. They also observed a 5.2-fold increased risk of glial tumors for the homozygous TT allele of *MTHFR* C677T; however, this increase was not statistically significant. Conversely, Salnikova and colleagues (2013) found no association of the *MTHFR* polymorphism and pediatric brain tumor risk [100]. Several studies have demonstrated an association between maternal folate intake (food or supplementation) during pregnancy and a reduced risk of brain tumors in children [101,102]. Bunnin and colleagues (1993) observed a reduced risk of PNET, but not astrocytoma associated with folate intake in young children [103]. Some Asian and African countries have struggled to provide the proper coverage of iron-folic acid supplementation during pregnancy, as recommended by WHO guidelines [104,105]. Therefore, the divergence observed between the studies could be associated with the maternal diet because folate and vitamins B6 and B12 are cofactors for SAM production and required for proper enzymatic activity. An appropriate diet might compensate for the reduced enzymatic activity conferred by *MTHFR* polymorphisms, reducing tumor risk.

The geographical distribution of folate-rich nutrients may be associated with the *MTHFR* C677T polymorphism frequency. Populations with a folate-rich diet have a higher frequency of the *MTHFR* 677TT genotype. This genotype has been associated with fetal survival, although it has also been associated with increased cancer risk. The *MTHFR* 677TT genotype has been associated with a protective role in adequate folate concentrations; nonetheless, it can enhance cancer risk when the folate supply is inadequate [106]. Therefore, folate and vitamin B6 and B12 levels should be included in *MTHFR* polymorphism studies in cancer risk assessment. One literature report observed no significant difference in the serum folic acid and vitamin B12 levels concerning *MTHFR* polymorphisms [107]. Additionally, they found no association between meningioma or glioma risk and the *MTHFR* C677T polymorphism in the Indian population. This literature report supports that the *MTHFR* C677T polymorphism does not increase the risk of cancer development when there is adequate folate intake.

Folate supplementation in animal models has a chemopreventive effect on the initial development of lesions in the colon. However, after establishing preneoplastic lesions, folate favors increased tumor growth, likely due to folate-dependent DNA synthesis in tumor cells [10,108,109]. Using rodent models of gliomagenesis, treatment with folate limits the development of glioma, inhibits tumor growth, and reduces overall methylation gain. Furthermore, folate supplementation reduces the methylation levels in tumor suppressor genes such as PTEN, P53 and Bax while increases the methylation levels of the MGMT promoter and oncogenes such as PDGF-B [108]. However, folic acid favors glioblastoma cell line stemness via tumor sphere formation, demonstrating *MTHFR* upregulation and hypomethylation compared with monolayers [110]. Folate may play a dual role in gliomas by preventing tumor initiation or promoting tumor progression.

Several studies have found no association between polymorphisms and glioma development [100,107,111] and overall survival [112]. Notably, the influence of ethnicity in *MTHFR* polymorphisms is associated with glioma risk. The genotypic frequency of *MTHFR* polymorphisms differs among ethnicities and can be a bias source in risk assessment [113]. Therefore, studies were addressed by population. An Indian study found no association of the *MTHFR* 677TT genotype with glioma, even when stratified by histological type or WHO grade [112]. Additionally, a Brazilian population-based study did not find the *MTHFR* 677TT polymorphism in astrocytic tumors, suggesting a protective role for this genotype [114]. The authors also stratified samples by histological grade (I–IV), but no significant difference was observed between the *MTHFR* 677CC and 677CT genotypes. The lack of significance could be associated with the small sample size in each stratification group. Conversely, a case-control study in Turkey with 74 patients and 94 controls demonstrated a higher frequency of the *MTHFR* 677TT genotype in high-grade gliomas than in the meningioma and control groups [115]. Brazilian and Turkish general populations have similar *MTHFR* 677TT polymorphism frequencies (7.7% and 7.1%, respectively), while the Indian population has a lower frequency (3.8–5%) [107,112,114,115]. These differences highlight the importance of studying diverse ethnic groups and stratifying glioma subtypes to perform more accurate analyses. Gliomas have different histological and molecular characteristics that should be grouped separately. The Turkish study evaluated high-grade gliomas, while the Brazilian study included only astrocytomas. The lack of studies with appropriate stratification is a limitation and might be partially responsible for the controversial literature. However, it is difficult to evaluate polymorphisms in stratified histological types because of the low incidence, as a result of the small number of patients per histological group.

Systematic reviews and meta-analyses can be useful tools to address the impact of publications with small sample sizes in the field. Two meta-analyses demonstrated no enhanced risk of glioma development based on the *MTHFR* C667T polymorphism [116,117]. One of them considered only case-control studies with gliomas (1786 cases and 2076 controls), and the other considered gliomas and meningiomas (3059 cases and 3324 controls). Carriers of *MTHFR* TC present an increased risk of meningioma [117]. According to ethnicity, the Asian group with *MTHFR* C677T presented a statistically non-significant higher risk of brain tumors, likely due to the small sample size. However, Caucasians showed no difference [116,117].

A meta-analysis was conducted to evaluate the impact of the *MTHFR* A1298C polymorphism on meningioma and glioma risk [118]. The analyses included 5 case-control studies with 2997 cases and 3403 controls. They found that heterozygous (AC versus AA) and dominant (CC + AC versus AA) variants were associated with an increased risk of meningiomas and gliomas, although only gliomas presented an increased risk associated with any *MTHFR* A1298C genotype. Furthermore, an increased risk was observed in the Caucasian population but not in the Asian population. However, an Indian population-based study demonstrated that the CC genotype had a 38% reduced chance of meningioma compared with the AA genotype of the *MTHFR* A1298C polymorphism [107]. Additionally, no enhanced risk was found when other SNPs were combined, such as *MTHFR* C677T and polymorphisms in *MTRR* and *MTR*.

In the Chinese population, two studies presented opposing results on meningioma risk. One study observed that meningiomas have a lower frequency of *MTHFR* 677TT and the T allele [119], while the other observed a significantly higher frequency [120]. Both reports demonstrated no difference in the *MTHFR* A1298C polymorphism. Additionally, no significant differences were found after WHO grade stratification [119,120]. A meta-analysis including 1615 meningioma cases and 1909 controls from nine case-control studies observed that the *MTHFR* 677CT genotype was associated with an increased risk of meningioma [121]. Stratification by population demonstrated no difference in Asian population, but an increased risk was associated with the Caucasian population. Similarly, the *MTHFR* A1298C polymorphism was associated with an increased meningioma risk only in the Caucasian population [122].

Meningiomas and gliomas are the most studied CNS tumors regarding *MTHFR* polymorphisms. A large case-control study with 1005 glioma cases, 631 meningioma cases and 1098 controls demonstrated that the heterozygosity for *MTHFR* A1298C and *MTHFR* C677T was associated with an increased risk of meningioma [10]. Glioma risk was increased only by *MTHFR* A1298C heterozygosity. Additionally, the authors stratified samples into GBM, oligodendrocytes, other astrocytomas and other glioma subtypes. They demonstrated that the *MTHFR* A1298C polymorphism was mostly associated with GBM and oligodendrocytes increased the risk. Regarding GBM, another study found no association between the *MTHFR* C677T polymorphism and disease risk [111]. Linnebank and colleagues demonstrated that the T-allele of the *MTHFR* C677T polymorphism is associated with poorer overall survival in GBM, likely mediated by reduced enzymatic activity [123]. The *MTHFR* c.677TT genotype is an independent poor prognostic factor in the younger patient group (<60 years). Reduced enzymatic activity has also been associated with the MGMT promoter and DNA hypomethylation [124]. Additionally, a retrospective cohort of patients with recurrent GBM presented genomic DNA hypomethylation [125]. The authors demonstrated that the *MTHFR* 677TT genotype was associated with reduced DNA methylation and suggested that patients harboring the *MTHFR* 677TT could benefit from treatment with a new approach based on perillyl alcohol inhalation.

Cadieux and colleagues (2006) evaluated the association of the *MTHFR* C677T polymorphism with DNA hypomethylation in GBM cell lines and patient samples [124]. All the cell lines evaluated and 8 of 10 primary GBM samples presented genome-wide hypomethylation. *MTHFR* is located at Chr1p, a region commonly deleted in GBM. Hence, four GBM samples presented allelic loss of *MTHFR*. The loss of the *MTHFR* allelic region and presence of low-functioning-allelic (CT and TT) genotypes were associated with a lower DNA methylation profile. Thus, only tumors with a CC genotype (Ala/Ala) presented no genomic hypomethylation. Collectively, these studies point to a central role of the *MTHFR* genotype in DNA hypomethylation in GBM. However, the impact of this phenomenon on glioma risk requires further confirmation.

In the present review, we detailed studies with controversial results regarding the impact of *MTHFR* polymorphisms on glioma risk. Herein, we discussed the following factors that might contribute to the controversial literature: a) ethnicity; b) histological subtypes; and c) dietary intake of vitamins B6 and B12 and folate. Despite the effort of several studies to address the ethnic impact in the risk assessment of *MTHFR* polymorphisms, small patient samples have limited the results. Additionally, histological stratification has limited statistical power due to the small sample size. A comprehensive evaluation in a larger cohort is essential to elucidate the influence of these factors on the relationship between *MTHFR* polymorphisms and glioma risk.

**Table 2:** Effect of the polymorphisms on brain tumor risk.

Study Type	Number of Participants	Ethnicity	SNP	Histological Stratification	Risk Association	Reference
Case-control Pediatric tumors	284 cases and 464 controls	Caucasian, East Slav and Russian (grouped together)	C677T	Glial tumors and embryonic brain tumors	No risk associated with brain tumor development	[100]
Case-control Pediatric tumors	73 cases and 205 controls	Thai	C677T	Brain tumors No stratification	Statistically non-significant 5.2 times increased risk of glial tumors for the homozygous TT allele	[99]
			A1298C		Increased risk of embryonic tumors No risk associated with glial and germ cell tumors	
Case-control Hospital based	108 gliomas, 76 meningiomas and 104 controls	Indian	C677T	No WHO malignancy grade stratification	No risk associated with gliomas or meningioma development	[107]
			A1298C		Increased risk of glioma 38% reduced risk of meningioma for CC and ‘C’ allele containing genotypes	
Case-control	112 glioma cases and 141 controls	Indian	C677T	Astrocytoma, glioblastoma, oligodendroglioma and other types of glioma	No risk associated with any glioma type or overall survival	[112]
Case-control Hospital based	39 HGGs, 35 meningiomas and 98 controls	Turkish	C677T	High-grade gliomas (HGG) and meningiomas	No risk associated with meningioma Non-significant 2.15 times increased risk of HGG for the homozygous TT allele	[115]
Case-control	93 cases and 93 controls	mixed Brazilian	C677T	Astrocytic tumors subdivided in WHO grade I (17), grade II (19), grade III (14), and grade IV (43)	Potential protective effect for the homozygous TT genotype No risk associated with histological subtypes	[114]
Case-control	6oo cases and 600 controls	Chinese Han population	C677T	Meningiomas subdivided in WHO grade I (391), grade II (167) and grade III (42)	Reduced risk associated with TT and ‘T’ allele-containing genotypes No risk associated with subtypes	[119]
			A1298C		No association with meningioma general risk or subtypes	
Case-control	317 cases and 320 controls	Northern Chinese Han population	C677T	Meningioma without WHO grade stratification	Increased risk of meningioma for the TT genotype	[120]
			A1298C		No risk association	
Case-control	631 meningioma, 1005 glioma and 1098 controls	Caucasian	C677T	GBM, oligodendrocytes, other astrocytomas and other gliomas’ subtypes	Increased risk of meningioma, but not glioma	[10]
			A1298C		Increased risk of meningioma, glioblastoma and oligodendroglioma for the heterozygous genotype	
Meta-analysis	1323 cases and 1883 controls from 10 studies	Caucasian, Chinese, Asian	C677T	Meningioma	No risk association	[122]
	1855 cases and 3331 controls		A1298C		Increased risk for Caucasian populations in heterozygous (AC) and dominant (CC + AC) models	
Meta-analysis	1615 cases and 1909 controls	Asian and Caucasian	C677T	Meningioma without WHO grade stratification	Increased meningioma risk for CT genotype carriers in the total population. No risk associated with Asian populations and increased risk for Caucasian populations with CT and TT genotypes	[121]
Meta-analysis	1786 cases and 2076 controls	Asian, Brazilian and Caucasian	C677T	Glioma without WHO grade stratification	No association was observed for total population or Caucasian populations	[116]
Meta-analysis	3059 cases and 3324 controls	Asian, Brazilian and Caucasian	C677T	Glioma and meningioma	Increased risk for T allele carriers (TC + TT) and 1.38 times increased risk of meningioma for TC carriers Asian populations had an increased brain tumor risk, but no association was observed for Caucasian populations	[117]
Meta-analysis	2236 cases and 2248 controls from five studies	Asian and Caucasian	A1298C	Glioma and meningioma	Increased glioma risk in the total population. In Caucasian populations, increased risk of meningioma and glioma in the heterozygous model (AC) and dominant model (CC + AC)	[118]
Case-control	328 cases and 400 controls	Caucasian	C677T	Glioblastoma	No risk associated with the polymorphism	[111]
Retrospective cohort study	214 patients	Caucasian	C677T	Glioblastoma	Poor overall survival in patients younger than 60 years	[123]
			A1298C		No association with overall survival	

PNET = primitive neuroectodermal tumor; AT/RT = atypical teratoid rhabdoid tumor; WHO = World Health Organization; HGG = high-grade glioma.

## 6. *MTHFR* C677T and A1298C Polymorphisms and Gastric Cancer

Gastric cancer (GC) is the fifth most common cancer and third leading cause of cancer-related death, responsible for almost 800,000 deaths each year [126]. Generally, the average age of its occurrence is 60–80 years, and cases younger than 30 years are rare. Thus, GC is considered a senile disease [127,128]. East Asian countries, followed by Eastern Europe and South American countries, have the highest incidence rates. The lowest rates are observed in North America and Africa [129]. Although the incidence of GC has decreased in most countries and despite advances in diagnoses, the disease is usually detected in advanced stages, mainly because of the nonspecificity of symptoms in early stages. The average survival in five years is only 20%; additionally, surgery and chemotherapy have limited value in treating advanced cases. Furthermore, few targeted therapies are available that use molecular markers [127].

GC is a multifactorial disease comprising lifestyle, aging, socioeconomic factors, infectious agents such as *Helicobacter pylori* (classified as group 1 carcinogenic and associated with 80% of cases), Epstein–Barr virus (associated with 10% of tumors) and multiple genetic and epigenetic alterations [128,130]. According to Lauren’s classification, gastric adenocarcinomas are basically divided into two types: intestinal (well differentiated with cohesive neoplastic cells, forming structures similar to tubular glands) and diffuse (poorly differentiated with infiltration and thickening of the stomach wall without a formation discrete mass). These two types differ not only under histological analysis but are also associated with sex, age, and other epidemiological characteristics [127,131,132].

Recently, new molecular classifications of GC have become available. The Cancer Genome Atlas (TCGA), using high-throughput technologies, has molecularly characterized gastric adenocarcinoma into four subtypes: Epstein–Barr virus (EBV)-infected tumors, microsatellite instability tumors (MSI), genomically stable tumors (GS), and chromosomally unstable tumors (CIN) [133]. Similarly, the Asian Cancer Research Group (ACRG) has characterized gastric adenocarcinoma into four subtypes: tumors with microsatellite stable/epithelial-to-mesenchymal transition (MSS/EMT), tumors with microsatellite instability (MSI), microsatellite stable/TP53 activity (MSS/TP53+) tumors, and microsatellite stable/TP53 inactivity (MSS/TP53-) tumors [133,134]. These initiatives have valuable potential in clinical implications, mainly in therapeutic development, new clinical trials and targeting.

The relationship between the *MTHFR* polymorphism and clinical outcomes of GC patients has been determined in many populations; however, the results remain controversial to some degree, demonstrating that the association between the methylenetetrahydrofolate reductase polymorphism and risk of GC is a complex issue. The relationship between the presence of C677T and A1298C polymorphisms and risk of GC among different populations is shown in Table 3.

A prospective study performed with an eastern Turkish population assessed the relationship between the *MTHFR* A1298C and C677T polymorphisms in gastrointestinal tumor development and included 70 GC patients. Individuals carrying genotype AC of the A1298C polymorphism compared with those with AA had a 4.13 times higher risk (*p* = 0.001) of developing GC, and individuals with the CC genotype compared with the AA genotype had a 2.91 times higher risk (*p* = 0.027). However, the risk of developing GC in individuals carrying the TT genotype of the C677T polymorphism compared with those with CC was not higher; regarding the genotypes, no difference was found in the life spans of the patients [135]. Recently, in a case (*n* = 307) control (*n* = 560) study in a Chinese Han population, patients carrying the TT genotype of C677T polymorphism were associated with a decreased risk of GC in older individuals and patients who never drank alcohol [136] Moreover, the relationship of the *MTHFR* C677T polymorphism and the combined use of alcohol and smoking have a coordinate effect influencing GC risk [137]. However, a meta-analysis of 5757 cases and 8501 controls of Asian and Caucasian patients found different results: a significant association was found between the GC and *MTHFR* C677T polymorphisms (homozygous model [TT vs. CC]: OR, 1.39; 95% CI, 1.20–1.62; heterozygous model [CT vs. CC]: OR, 1.18; 95% CI, 1.05–1.32; dominant model [TT + CT vs. CC]: OR, 1.23; 95% CI, 1.10–1.38; recessive model [TT vs. CC + CT]: OR, 1.26; 95% CI, 1.12–1.42) [138]. Further investigations also indicated an elevated risk of GC in Asian individuals carrying the *MTHFR* C677T polymorphism but not in Caucasian populations [138]. Similar results were found by Zintzaras and colleagues [139]. Additionally, in an Italian study evaluating 790 CG patients and 202 healthy controls, the TT genotype was associated with an increased risk of GC with ORs of 1.52 (log-additive model of inheritance) and 2.35 (codominant model of inheritance) [140]. Similar results were found in a study of genetic susceptibility to cancer involving GC samples (*n* = 2727), where *MTHFR* C677T was associated with GC (TT vs. CT + CC; OR: 1.52, 95% CI: 1.31–1.77, *p*-value = 4.9 × 10^−8^) [141].

In a meta-analysis involving 6572 cases and 9584 controls comprising Caucasian, Asian and mixed ethnicity samples, the *MTHFR* C677T polymorphism significantly increased the susceptibility to GC, but no significant correlation was found with the A1298C polymorphism. Furthermore, the authors found that *MTHFR* C677T but not *MTHFR* A1298C was associated with an increased risk of GC in Asian and Caucasian populations [142]. Another divergent result was found in a meta-analysis performed by Dong and colleagues [143], who evaluated 4070/6462 cases/controls for C677T and 1923/3561 cases/controls for the A1298C polymorphism. No significant association was found in the CC genotype of A1298C; however, the C677T allele T compared with allele C was associated with a 17.3% increased risk. Additionally, subgroup analyses revealed an increased risk to Asian populations but not to Caucasian populations [143]. Furthermore, a study of *MTHFR* C677T and GC susceptibility in a population (*n* = 167) from the Ardabil Province in Iran found that CT heterozygous individuals had a lower susceptibility to GC. Additionally, CT was correlated with a reduced risk in female and older participants [144]. Similar results were found in Korean [145] and Mexican studies [146].

*MTHFR* polymorphisms also play important roles in the response of GC patients to treatments. In a meta-analysis involving seven case-control studies and six cohort studies (*n* = 1718), Tang and colleagues evaluated the impact of the C677T polymorphism in GC patients treated with 5-fluorouracil (5-FU)-based chemotherapy [147]. 5-FU or its enhanced version, S-1, is a standard therapy for GC patients, alone or in combination with other agents [148]. No association was found between the C677T polymorphism and response rate [TT/(CC + CT) OR = 1.31, 95% CI: 0.62–2.76] or overall survival [(CT + TT)/CC HR = 1.05, 95% CI: 0.86–1.26; TT/(CT + CC) HR = 1.48, 95% CI: 0.53–4.15]. However, individuals who carry the TT genotype tended to present more hematologic toxicity than those with the CC or CT genotype [(CC + CT)/TT OR = 0.66, 95% CI: 0.48–0.91]. MTHFR is associated with 5-FU metabolism [149], and this result may be explained by the reduction in MTHFR activity caused by the TT genotype, leading to efficient inhibition of thymidylate synthase (TS) and increased 5-FU efficiency, which may induce more toxicity to patients because 5-FU is a cytotoxic agent [147]. Other GC treatments seem to be influenced by *MTHFR* polymorphisms. In the Netherlands, a multicenter phase 2 study investigated the use of bevacizumab combined with docetaxel, oxaliplatin, and capecitabine as a first-line treatment for advanced HER2-negative GC patients and found that the *MTHFR* C677T polymorphism was related to outcomes in which patients carrying the TT genotype had inferior progression-free survival (vs CC/CT: HR, 4.7; 95% CI, 1.75–12.8 [*p* = 0.0007]) and OS (vs CC/CT: HR, 5.9; 95% CI, 2.12–16.5 [*p* = 0.0001] [150]. Additionally, in a study of a German population, the *MTHFR* A1298C polymorphism was an independent prognostic factor associated with a poor prognosis in neoadjuvantly treated GC patients [151].

*MTHFR* polymorphisms also impact the severity of atrophic gastritis, which is a GC precursor lesion. *Helicobacter pylori*-negative patients carrying the TT genotype showed an increased risk of moderate-to-severe lesions; thus, *MTHFR* C677T may act as a predictive factor for GC precancerous lesions [152]. Saberi and colleagues found an increased risk of GC in a case (*n* = 450) control (*n* = 780) study of an Iranian population in patients carrying the *MTHFR* C677T polymorphism who were also positive for *Helicobacter pylori* infection [153]. The TT genotype of MTHFR was more likely to be associated with H. pylori infection than CC and CT genotypes, in a Chinese population of GC patients, and may be considered as a susceptible factor of a risk to H. pylori infection [154].

As discussed above, the effect of the *MTHFR* polymorphisms in GC is not homogeneous among the populations. This complex variation may be caused by the interaction of genetic and environmental factors, such as variant alleles, dietary habits, smoking, folate intake, alcohol consumption, *Helicobacter pylori* infection, and the molecular background specific to each population. Thus, it is difficult to draw conclusions about *MTHFR* polymorphisms and the risk of GC.

**Table 3:** Effect of the polymorphisms on gastric cancer risk.

Study Type	Number of Participants	Ethnicity	SNP	Risk Association	References
Case-control	70 GC and 61 controls	Turkish	C677T A1298C	The AC genotype of the A1298C polymorphism is a risk factor for GC. The TT genotype of C677T was not at a higher risk than the CC genotype.	[135]
Case-control	307 GC and 560 controls	Chinese	C677T	TT genotype associated with a decreased risk for GC	[136]
Case-control	107 GC and 220 controls	Chinese	C677T	*MTHFR* variant genotypes + smoking and drink habits are associated with a higher risk for GC	[137]
Meta-analysis	5757 GC and 8501 controls	Asian and Caucasian	C677T	Significant association was found between GC and the *MTHFR* C677T polymorphism. Elevated risk of GC in Asian individuals carrying the *MTHFR* C677T polymorphism, but not in Caucasian populations	[138]
Meta-analysis	1584 GC and 2785 controls	Asian and Caucasian	C677T A1298C	*MTHFR* C677T and A1298C polymorphisms, respectively, contribute to the susceptibility of GC. In East Asian populations with C677T, the association was significant but not in Caucasian populations. The A1298C polymorphism was associated with GCA in East Asian populations.	[139]
Case-control	790 GC and 202 controls	Italian	C677T A1298C	Increased risk of GC for the C677T variant (homozygous TT), but no effect of the A1298C polymorphism.	[140]
Meta-analysis	2727	Asian, Caucasian and Mixed	C677T	*MTHFR* C677T was associated with GC	[141]
Meta-analysis	6572 GC and 9584 controls	Asian, Caucasian and Mixed	C677T A1298C	C677T was related to a significantly increased risk for GC. No correlation was found with A1298C. C677T, but not A1298C, was associated with an increased risk of GC in Asian and Caucasian populations	[142]
Meta-analysis	4070/6462 cases/controls for C677T and 1923/3561 cases/controls for A1298C polymorphism	Eastern and Western	C677T A1298C	No significant association was found in the CC genotype of A1298C. The C677T allele T was associated with an increased risk of GC. Subgroup analyses revealed an increased risk for Asian populations but not for Caucasian populations	[143]
Case-control	76 GC and 91 controls	Turkish	C677T	CT heterozygotes had a lower susceptibility to GC	[144]
Meta-analysis	1718	Asian, European and Mixed	C677T	TT was related more to hematologic toxicity than the CC or CT genotype	[147]
Multicenter, single-arm, phase 2 study	60	Dutch	C677T	The TT genotype was related to inferior progression-free survival and OS	[150]
Retrospective comparative exploratory study	218	German	C677T A1298C	A1298C was an independent prognostic factor associated with a poor prognosis in neoadjuvantly treated GC patients	[151]
Single-center, cross sectional observational trial	128	Chinese	C677T	The TT genotype showed an increased risk of moderate-to-severe precancerous gastric lesions	[152]
Case-control study	450 GC and 780 controls	Iranian	C677T	*MTHFR* C677T carriers who were also positive for *H. pylori*, showed an increased risk for GC	[153]
Case-control	58 GC patients with H. pylori infection and 94 non-infected patients	Chinese	C677T	TT genotype was considered a susceptibility factor of H. pylori infection.	[154]

## 7. Conclusions

In the present review, we detailed studies with controversial results regarding the impact of *MTHFR* polymorphisms on breast cancer, glioma and gastric cancer risk. In addition to the controversial literature, we observed a more relevant influence of the *MTHFR* C677T polymorphism, particularly in the Caucasian and Asian populations, on the risk of developing BC. However, the *MTHFR* A1298C polymorphism also showed relevance in some cases concerning the risk of breast cancer and relation to more aggressive phenotypes or lymphonodal metastases. *MTHFR* C677T polymorphisms are more associated with an increased meningioma risk, primarily in Caucasian populations. Regarding *MTHFR* A1298C polymorphisms, it appears to be more associated with glioma risk, mainly the heterozygous genotype. The effect of the *MTHFR* polymorphisms in GC is heterogeneous and may be associated with epigenetic and environmental factors.

In a metabolomic study performed using serum samples from Greek women who were BC controls, a significant interaction was observed between the *MTHFR* C677T polymorphism and a Mediterranean diet rich in fruits and vegetables and the modulation of the serum levels of the 5-MTHFR enzyme [155]. Another group evaluated gene-environment interactions and predictors of colorectal cancer and suggested that interactions of the environment would be related to *MTHFR* polymorphism and the prevention and/or induction of colorectal cancer [156]. Further studies relate to *MTHFR* polymorphisms and the mechanisms of DNA repair and metabolomic studies can better clarify the relationship of the influence of these polymorphisms on the risk of BC, glioma and CG. Additionally, comprehensive evaluation in a larger cohort stratified by ethnicity, histological subtype and considering vitamins B6 and B12 and folate levels is crucial to further understand the association of *MTHFR* polymorphisms and BC and glioma and CG risk.

## References

[1] Berry R.J., Bailey L., Mulinare J., Bower C., Dary O. (2010). Fortification of Flour with Folic Acid. Food Nutr. Bull..

[2] Ebara S. (2017). Nutritional role of folate. Congenit. Anom..

[3] Salazar M.D., Ratnam M. (2007). The folate receptor: What does it promise in tissue-targeted therapeutics?. Cancer Metastasis Rev..

[4] Stover P.J. (2009). One-Carbon Metabolism–Genome Interactions in Folate-Associated Pathologies. J. Nutr..

[5] Bae S., Ulrich C.M., Bailey L.B., Malysheva O., Brown E.C., Maneval D.R., Neuhouser M.L., Cheng T.Y.D., Miller J.W., Caudill M.A. (2014). Impact of folic acid fortification on global DNA methylation and one-carbon biomarkers in the Women’s Health Initiative Observational Study cohort. Epigenetics.

[6] Bailey L.B., Stover P.J., McNulty H., Fenech M.F., Gregory J.F., Mills J.L., Pfeiffer C.M., Fazili Z., Zhang M., Ueland P.M. (2015). Biomarkers of Nutrition for Development—Folate Review. J. Nutr..

[7] Duthie S.J., Narayanan S., Brand G.M., Pirie L., Grant G. (2002). Impact of Folate Deficiency on DNA Stability. J. Nutr..

[8] Donnelly J.G. (2001). Folic Acid. Crit. Rev. Clin. Lab. Sci..

[9] Berger S.H., Pittman D.L., Wyatt M.D. (2008). Uracil in DNA: Consequences for carcinogenesis and chemotherapy. Biochem. Pharm..

[10] Bethke L., Webb E., Murray A., Schoemaker M., Feychting M., Lönn S., Ahlbom A., Malmer B., Henriksson R., Auvinen A. (2008). Functional Polymorphisms in Folate Metabolism Genes Influence the Risk of Meningioma and Glioma. Cancer Epidemiol. Biomark. Prev..

[11] Hasan T., Arora R., Bansal A.K., Bhattacharya R., Sharma G.S., Singh L.R. (2019). Disturbed homocysteine metabolism is associated with cancer. Exp. Mol. Med..

[12] Bravatà V. (2014). Controversial roles of methylenetetrahydrofolate reductase polymorphisms and folate in breast cancer disease. Int. J. Food Sci. Nutr..

[13] Matsuo K., Ito H., Wakai K., Hirose K., Saito T., Suzuki T., Kato T., Hirai T., Kanemitsu Y., Hamajima H. (2005). One-carbon metabolism related gene polymorphisms interact with alcohol drinking to influence the risk of colorectal cancer in Japan. Carcinogenesis.

[14] Singal R., Ferdinand L., Das P.M., Reis I.M., Schlesselman J.J. (2004). Polymorphisms in the methylenetetrahydrofolate reductase gene and prostate cancer risk. Int. J. Oncol..

[15] Robien K., Ulrich C.M. (2003). 5,10-Methylenetetrahydrofolate reductase polymorphisms and leukemia risk: A HuGE minireview. Am. J. Epidemiol..

[16] Hickey S.E., Curry C.J., Toriello H.V. (2013). ACMG Practice Guideline: Lack of evidence for MTHFR polymorphism testing. Genet. Med..

[17] Tsang B.L., Devine O.J., Cordero A.M., Marchetta C.M., Mulinare J., Mersereau P., Guo J., Qi Y.P., Berry R.J., Rosenthal J. (2015). Assessing the association between the methylenetetrahydrofolate reductase (MTHFR) 677C>T polymorphism and blood folate concentrations: A systematic review and meta-analysis of trials and observational studies. Am. J. Clin. Nutr..

[18] De Mattia E., Toffoli G. (2009). C677T and A1298C MTHFR polymorphisms, a challenge for antifolate and fluoropyrimidine-based therapy personalisation. Eur. J. Cancer.

[19] Farkas S.A., Böttiger A.K., Isaksson H.S., Finnell R.H., Ren A., Nilsson T.K., Nilsson T.K. (2013). Epigenetic alterations in folate transport genes in placental tissue from fetuses with neural tube defects and in leu-kocytes from subjects with hyperhomocysteinemia. Epigenetics.

[20] Toffoli G., De Mattia E. (2008). Pharmacogenetic relevance of MTHFR polymorphisms. Pharmacogenomics.

[21] Esteller M. (2008). Epigenetics in cancer. N. Engl. J. Med..

[22] Semmler A., Simon M., Moskau S., Linnebank M. (2008). Polymorphisms of methionine metabolism and susceptibility to meningioma formation: Laboratory investigation. J. Neurosurg..

[23] Ulrich C.M. (2005). Nutrigenetics in Cancer Research—Folate Metabolism and Colorectal Cancer. J. Nutr..

[24] Goyette P., Pai A., Milos R., Frosst P., Tran P., Chen Z., Chan M., Rozen R. (1998). Gene structure of human and mouse methylenetetrahydrofolate reductase (MTHFR). Mamm. Genome.

[25] Goyette P., Rozen R. (2000). The thermolabile variant 677C?T can further reduce activity when expressed in CIS with severe mutations for human methylenetetrahydrofolate reductase. Hum. Mutat..

[26] Levin B.L., Varga E. (2016). MTHFR: Addressing Genetic Counseling Dilemmas Using Evidence-Based Literature. J. Genet. Couns..

[27] Yang Q.-H., Botto L.D., Gallagher M., Friedman J.M., Sanders C.L., Koontz D., Nikolova S., Erickson J.D., Steinberg K. (2008). Prevalence and effects of gene-gene and gene-nutrient interactions on serum folate and serum total homocysteine concentrations in the United States: Findings from the third National Health and Nutrition Examination Survey DNA Bank. Am. J. Clin. Nutr..

[28] Rosenberg N., Murata M., Ikeda Y., Opare-Sem O., Zivelin A., Geffen E., Seligsohn U. (2002). The frequent 5,10-methylenetetrahydrofolate reductase C677T polymorphism is associated with a common haplo-type in whites, Japanese, and Africans. Am. J. Hum. Genet.

[29] Kauwell G.P., Wilsky C.E., Cerda J.J., Herrlinger-Garcia K., Hutson A.D., Theriaque D.W., Boddie A., Rampersaud G.C., Bailey L.B. (2000). Methylenetetrahydrofolate reductase mutation (677C → T) negatively influences plasma homocysteine response to marginal folate intake in elderly women. Metabolism.

[30] Rozen R. (1997). Genetic Predisposition to Hyperhomocysteinemia: Deficiency of Methylenetetrahydrofolate Reductase (MTHFR). Thromb. Haemost..

[31] Frosst P., Blom H., Milos R., Goyette P., Sheppard C., Matthews R., Boers G., Heijer M.D., Kluijtmans L., Heuve L.V.D. (1995). A candidate genetic risk factor for vascular disease: A common mutation in methylenetetrahydrofolate reductase. Nat. Genet..

[32] Ulrich C.M., Robien K., Sparks R. (2002). Pharmacogenetics and folate metabolism –a promising direction. Pharmacogenomics.

[33] Lievers K.J., Boers G.H., Verhoef P., Heijer M., Kluijtmans L.A., Put N.M., Trijbels F.J., Blom H.J. (2001). A second common variant in the methylenetetrahydrofolate reductase (MTHFR) gene and its relationship to MTHFR enzyme activity, homocysteine, and cardiovascular disease risk. J. Mol. Med..

[34] Friedman G., Goldschmidt N., Friedlander Y., Ben-Yehuda A., Selhub J., Babaey S., Mendel M., Kidron M., Bar-On H. (1999). A Common Mutation A1298C in Human Methylenetetrahydrofolate Reductase Gene: Association with Plasma Total Homocysteine and Folate Concentrations. J. Nutr..

[35] Cao Y., Xu J., Zhang Z., Huang X., Zhang A., Wang J., Zheng Q., Fu L., Du J. (2013). Association study between methylenetetrahydrofolate reductase polymorphisms and unexplained recurrent pregnancy loss: A meta-analysis. Gene.

[36] Wu W., Shen O., Qin Y., Lu J., Niu X., Zhou Z., Lu C., Xia Y., Wang S., Wang X. (2011). Methylenetetrahydrofolate reductase C677T polymorphism and the risk of male infertility: A meta-analysis. Int. J. Androl..

[37] Kang S., Zhao X., Liu L., Wu W., Zhang N. (2013). Association of the C677T Polymorphism in the MTHFR Gene with Hemorrhagic Stroke: A Meta-Analysis. Genet. Test. Mol. Biomark..

[38] Zhu B., Wu X., Zhi X., Zheng Q., Sun G. (2014). Methylenetetrahydrofolate reductase C677T polymorphism and type 2 diabetes mellitus in Chinese population: A me-ta-analysis of 29 case-control studies. PLoS ONE.

[39] Chang W.-W., Zhang L., Yao Y.-S., Su H., Jin Y.-L., Chen Y. (2013). Methylenetetrahydrofolate reductase (MTHFR) C677T polymorphism and susceptibility to diabetic nephropathy in Chinese type 2 diabetic patients: A meta-analysis. Ren. Fail..

[40] Karabacak E., Aydin E., Ozcan O., Dogan B., Gultepe M., Cosar A., Muftuoglu T. (2014). Methylenetetrahydrofolate reductase (MTHFR) 677C>T gene polymorphism as a possible factor for reducing clini-cal severity of psoriasis. Int. J. Clin. Exp. Med..

[41] Peng Q., Lao X., Huang X., Qin X., Li S., Zeng Z. (2015). The MTHFR C677T polymorphism contributes to increased risk of Alzheimer’s Disease: Evidence based on 40 case-control studies. Neurosci. Lett..

[42] Ray J., Shmorgun D., Chan W. (2002). Common C677T Polymorphism of the Methylenetetrahydrofolate Reductase Gene and the Risk of Venous Thromboembolism: Meta-Analysis of 31 Studies. Pathophysiol. Haemost. Thromb..

[43] McColgan P., Sharma P. (2008). The Genetics of Carotid Dissection: Meta-Analysis of a MTHFR/C677T Common Molecular Variant. Cereb. Dis..

[44] Hu C.-Y., Qian Z.-Z., Gong F.-F., Lu S.-S., Feng F., Wu Y.-L., Yang H.-Y., Sun Y.-H. (2014). Methylenetetrahydrofolate reductase (MTHFR) polymorphism susceptibility to schizophrenia and bipolar disorder: An updated meta-analysis. J. Neural. Transm..

[45] Liew S.C., Gupta E.D. (2015). Methylenetetrahydrofolate reductase (MTHFR) C677T polymorphism: Epidemiology, metabolism and the associated diseases. Eur. J. Med. Genet..

[46] Bolufer P., Barragán E., Collado M., Cervera J., Lopez J.-A., Sanz M.A. (2006). Influence of genetic polymorphisms on the risk of developing leukemia and on disease progression. Leuk. Res..

[47] Zanrosso C.W., Hatagima A., Emerenciano M., Ramos F., Figueiredo A., Félix T.M., Segal S.L., Giugliani R., Muniz M.T.C., Pombo-De-Oliveira M.S. (2006). The role of methylenetetrahydrofolate reductase in acute lymphoblastic leukemia in a Brazilian mixed population. Leuk. Res..

[48] Yan J., Yin M., Dreyer Z.E., Scheurer M.E., Kamdar K., Wei Q., Okcu M.F. (2012). A meta-analysis of MTHFR C677T and A1298C polymorphisms and risk of acute lymphoblastic leukemia in children. Pe-Diatr Blood Cancer.

[49] Kurzwelly D., Knop S., Guenther M., Loeffler J., Korfel A., Thiel E., Hebart H., Simon M., Weller M., Linnebank M. (2010). Genetic variants of folate and methionine metabolism and PCNSL incidence in a German patient population. J. Neuro-Oncol..

[50] Kim H.N., Lee I., Kim Y., Tran H.T.T., Yang D., Lee J., Shin M., Park K., Shin M., Choi J. (2008). Association between folate-metabolizing pathway polymorphism and non-Hodgkin lymphoma. Br. J. Haematol..

[51] Tang M., Wang S.-Q., Liu B.-J., Cao Q., Li B.-J., Li P.-C., Li Y.-F., Qin C., Zhang W. (2014). The methylenetetrahydrofolate reductase (MTHFR) C677T polymorphism and tumor risk: Evidence from 134 case–control studies. Mol. Biol. Rep..

[52] Wen Y.-Y., Yang S.-J., Zhang J.-X., Chen X.-Y. (2013). Methylenetetrahydrofolate reductase genetic polymorphisms and esophageal squamous cell carcinoma susceptibility: A meta-analysis of case-control studies. Asian Pac. J. Cancer Prev..

[53] Fang Y., Xiao F., An Z., Hao L. (2011). Systematic review on the relationship between genetic polymorphisms of methylenetetrahydrofolate reductase and esophageal squamous cell carcinoma. Asian Pac. J. Cancer Prev..

[54] Boccia S., Hung R., Ricciardi G., Gianfagna F., Ebert M.P.A., Fang J.-Y., Gao C.-M., Götze T., Graziano F., Lacasaña-Navarro M. (2007). Meta- and Pooled Analyses of the Methylenetetrahydrofolate Reductase C677T and A1298C Polymorphisms and Gastric Cancer Risk: A Huge-GSEC Review. Am. J. Epidemiol..

[55] Ulvik A., Evensen E.T., Lien E.A., Hoff G., Vollset S.E., Majak B.M., Ueland P.M. (2001). Smoking, folate and methylenetetrahydrofolate reductase status as interactive determinants of adenomatous and hyper-plastic polyps of colorectum. Am. J. Med. Genet..

[56] Friso S., Choi S.-W., Girelli D., Mason J.B., Dolnikowski G.G., Bagley P.J., Olivieri O., Jacques P.F., Rosenberg I.H., Corrocher R. (2002). A common mutation in the 5,10-methylenetetrahydrofolate reductase gene affects genomic DNA methylation through an interaction with folate status. Proc. Natl. Acad. Sci. USA.

[57] Stern L.L., Mason J.B., Selhub J., Choi S.W. (2000). Genomic DNA hypomethylation, a characteristic of most cancers, is present in peripheral leukocytes of individuals who are homozygous for the C677T polymorphism in the methylenetetrahydrofolate reductase gene. Cancer Epidemiol. Biomark. Prev..

[58] Quinlivan E.P., Davis S.R., Shelnutt K.P., Henderson G.N., Ghandour H., Shane B., Selhub J., Bailey L.B., Stacpoole P.W., Gregory J.F. (2005). Methylenetetrahydrofolate Reductase 677C→T Polymorphism and Folate Status Affect One-Carbon Incorporation into Human DNA Deoxynucleosides. J. Nutr..

[59] Cecilio A.P., Takakura E.T., Jumes J.J., Dos Santos J.W., Herrera A.C., Victorino V.J., Panis C. (2015). Breast cancer in Brazil: Epidemiology and treatment challenges. Breast Cancer Dove Med. Press.

[60] Ciriello G., Sinha R., Hoadley K.A., Jacobsen A.S., Reva B., Perou C.M., Sander C., Schultz N. (2013). The molecular diversity of Luminal A breast tumors. Breast Cancer Res. Treat..

[61] Network C.G.A. (2012). Comprehensive molecular portraits of human breast tumours. Nature.

[62] Goldhirsch A., Winer E.P., Coates A.S., Gelber R.D., Piccart-Gebhart M., Thürlimann B., Senn H.-J., Albain K.S., André F., Bergh J. (2013). Personalizing the treatment of women with early breast cancer: Highlights of the St Gallen International Expert Consensus on the Primary Therapy of Early Breast Cancer. Ann. Oncol..

[63] Petrone I., Rodrigues F.R., Fernandes P.V., Abdelhay E. (2020). Immunohistochemical Biomarkers in Ductal Carcinoma in Situ. Open J. Pathol..

[64] Kalemi T.G., Lambropoulos A.F., Gueorguiev M., Chrisafi S., Papazisis K.T., Kotsis A. (2005). The association of p53 mutations and p53 codon 72, Her 2 codon 655 and MTHFR C677T polymorphisms with breast cancer in Northern Greece. Cancer Lett..

[65] Larsson S.C., Giovannucci E., Wolk A. (2007). Folate and Risk of Breast Cancer: A Meta-analysis. J. Natl. Cancer Inst..

[66] Lu Q., Jiang K., Li Q., Ji Y.-J., Chen W.-L., Xue X.-H. (2015). Polymorphisms in the MTHFR gene are associated with breast cancer risk and prognosis in a Chinese population. Tumor Biol..

[67] Awwad N., Yousef A.-M., Abuhaliema A., Abdalla I., Yousef M. (2015). Relationship between Genetic Polymorphisms in MTHFR (C677T, A1298C and their Haplotypes) and the Incidence Of Breast Cancer among Jordanian Females—Case-Control Study. Asian Pac. J. Cancer Prev..

[68] Hesari A., MalekSabet A., Tirkani A.N., Ghazizadeh H., Iranifar E., Mohagheg F., Anoshrvani A.A., Ghasemi F. (2019). Evaluation of the two polymorphisms rs1801133 in MTHFR and rs10811661 in CDKN2A/B in breast cancer. J. Cell. Biochem..

[69] Gao C.-M., Tang J.-H., Cao H.-X., Ding J.-H., Wu J.-Z., Wang J., Liu Y.-T., Li S.-P., Su P., Matsuo K. (2009). MTHFR polymorphisms, dietary folate intake and breast cancer risk in Chinese women. J. Hum. Genet..

[70] Sohn K.-J., Croxford R., Yates Z., Lucock M., Kim Y.-I. (2004). Effect of the methylenetetrahydrofolate reductase C677T polymorphism on chemosensitivity of colon and breast cancer cells to 5-fluorouracil and methotrexate. J. Natl. Cancer Inst..

[71] Shrubsole M.J., Jin F., Dai Q., Shu X.O., Potter J.D., Hebert J.R., Gao Y.T., Zheng W. (2001). Dietary folate intake and breast cancer risk: Results from the Shanghai Breast Cancer Study. Cancer Res..

[72] He L., Shen Y. (2017). MTHFR C677T polymorphism and breast, ovarian cancer risk: A meta-analysis of 19,260 patients and 26,364 controls. Onco. Targets Ther..

[73] Pooja S., Carlus J., Sekhar D., Francis A., Gupta N., Konwar R., Kumar S., Kumar S., Thangaraj K., Rajender S. (2015). MTHFR 677C>T Polymorphism and the Risk of Breast Cancer: Evidence from an Original Study and Pooled Data for 28031 Cases and 31880 Controls. PLoS ONE.

[74] Li K., Li W., Dong X. (2014). Association of 677 C>T (rs1801133) and 1298 A>C (rs1801131) Polymorphisms in the MTHFR Gene and Breast Cancer Susceptibility: A Meta-Analysis Based on 57 Individual Studies. PLoS ONE.

[75] Meneses-Sanchez P., Garcia-Hernandez S.C., Porchia L.M., Pérez-Fuentes R., Torres-Rasgado E., Soto A.D.A., Gonzalez-Mejia M.E. (2019). C677T and A1298C methylenetetrahydrofolate reductase polymorphisms and breast cancer susceptibility among Latinos: A meta-analysis. Breast Cancer.

[76] Zara-Lopes T., Gimenez-Martins A., Nascimento-Filho C., Castanhole-Nunes M., Galbiatti-Dias A., Padovani-Júnior J., Maniglia J., Francisco J., Pavarino E., Goloni-Bertollo E. (2016). Role of MTHFR C677T and MTR A2756G polymorphisms in thyroid and breast cancer development. Genet. Mol. Res..

[77] He J., Pu Y., Wu Y., Qin R., Zhang Q., Sun Y., Zheng W., Chen L. (2014). Association between dietary intake of folate and MTHFR and MTR genotype with risk of breast cancer. Genet. Mol. Res..

[78] Jiang-Hua Q., De-Chuang J., Zhen-Duo L., Shu-De C., Zhenzhen L. (2014). Association of methylenetetrahydrofolate reductase and methionine synthase polymorphisms with breast cancer risk and interaction with folate, vitamin B6, and vitamin B12 intakes. Tumor Biol..

[79] Akilzhanova A., Nurkina Z., Momynaliev K., Ramanculov E., Zhumadilov Z., Zhumadilov Z., Rakhypbekov T., Hayashida N., Nakashima M., Takamura N. (2013). Genetic profile and determinants of homocysteine levels in Kazakhstan patients with breast cancer. Anticancer. Res..

[80] Diakite B., Tazzite A., Hamzi K., Jouhadi H., Nadifi S. (2012). Methylenetetrahydrofolate Reductase C677T polymorphism and breast cancer risk in Moroccan women. Afr. Health Sci..

[81] Rezende L.M., Marson F.A.L., Lima C.S.P., Bertuzzo C. (2017). Can MTHFR C677T and A1298C Polymorphisms Alter the Risk and Severity of Sporadic Breast Cancer in Bra-zilian Women?. Clin. Breast Cancer.

[82] Liu W., Li Y., Li R., Han X., Ma Y., Liu B., Kong X. (2016). Association of mthfr A1298C polymorphism with breast cancer and/or ovarian cancer risk: An updated me-ta-analysis. Afr. J. Tradit. Complement. Altern. Med..

[83] Durán M., Ángel C., Araújo M.D., Pinheiro D.D.R., Burbano R.M.R., Borges B.D.N. (2021). Thymidylate synthase and methylenetetrahydrofolate reductase polymorphisms and breast cancer susceptibility in a Brazilian population. Meta Gene..

[84] Castiglia P., Sanna V., Azara A., De Miglio M.R., Murgia L., Pira G., Sanges F., Fancellu A., Carru C., Bisail M. (2019). Methylenetetrahydrofolate reductase (MTHFR) C677T and A1298C polymorphisms in breast cancer: A Sardinian preliminary case-control study. Int. J. Med. Sci..

[85] Houghton S.C., Eliassen A.H., Zhang S.M., Selhub J., Rosner B.A., Willett W.C., Hankinson S.E. (2019). Plasma B-vitamins and one-carbon metabolites and the risk of breast cancer in younger women. Breast Cancer Res. Treat..

[86] Omran M.H., E Fotouh B., Shousha W.G., Ismail A., Ibrahim N.E., Ramadan S.S. (2021). Strong Correlation of MTHFR Gene Polymorphisms with Breast Cancer and its Prognostic Clinical Factors among Egyptian Females. Asian Pac. J. Cancer Prev..

[87] Ajaz S., Ali S.M., Siddiqa A., Memon M.A., Abid A., Khaliq S. (2021). Independent and Combined Associations of 677C/T and 1298A/C Polymorphisms in the MTHFR gene with Breast Cancers in a South-Asian Population. Medrxiv.

[88] Miranda-Filho A., Piñeros M., Soerjomataram I., Deltour I., Bray F. (2016). Cancers of the brain and CNS: Global patterns and trends in incidence. Neuro-oncology.

[89] Philips A., Henshaw D., Lamburn G., O’Carroll M. (2018). Brain Tumours: Rise in Glioblastoma Multiforme Incidence in England 1995–2015 Suggests an Adverse Environ-mental or Lifestyle Factor. J. Environ. Public Health.

[90] Bell J.S., Koffie R.M., Rattani A., Dewan M.C., Baticulon R.E., Qureshi M.M., Wahjoepramono E.J., Rosseau G., Park K., Nahed B.V. (2019). Global incidence of brain and spinal tumors by geographic region and income level based on cancer registry data. J. Clin. Neurosci..

[91] Leece R., Xu J., Ostrom Q.T., Chen Y., Kruchko C., Barnholtz-Sloan J.S. (2017). Global incidence of malignant brain and other central nervous system tumors by histology, 2003–2007. Neuro-Oncology.

[92] Ostrom Q.T., Cioffi G., Gittleman H., Patil N., Waite K., Kruchko C., Barnholtz-Sloan J.S. (2019). CBTRUS Statistical Report: Primary Brain and Other Central Nervous System Tumors Diagnosed in the United States in 2012–2016. Neuro-Oncology.

[93] Louis D.N., Ohgaki H., Wiestler O.D., Cavenee W.K., Burger P.C., Jouvet A., Scheithauer B.W., Kleihues P. (2007). The 2007 WHO Classification of Tumours of the Central Nervous System. Acta Neuropathol..

[94] Louis D.N., Perry A., Reifenberger G., Von Deimling A., Figarella-Branger D., Cavenee W.K., Ohgaki H., Wiestler O.D., Kleihues P., Ellison D.W. (2016). The 2016 World Health Organization Classification of Tumors of the Central Nervous System: A summary. Acta Neuropathol..

[95] Izmirli M. (2012). A literature review of MTHFR (C677T and A1298C polymorphisms) and cancer risk. Mol. Biol. Rep..

[96] Davis C.D., Uthus E.O. (2004). DNA Methylation, Cancer Susceptibility, and Nutrient Interactions. Exp. Biol. Med..

[97] Kulis M., Esteller M. (2010). DNA Methylation and Cancer. Adv. Genet..

[98] Van Tongelen A., Loriot A., De Smet C. (2017). Oncogenic roles of DNA hypomethylation through the activation of cancer-germline genes. Cancer Lett..

[99] Sirachainan N., Wongruangsri S., Kajanachumpol S., Pakakasama S., Visudtibhan A., Nuchprayoon I., Lusawat A., Phudhicharoenrat S., Shuangshoti S., Hongeng S. (2008). Folate pathway genetic polymorphisms and susceptibility of central nervous system tumors in Thai children. Cancer Detect. Prev..

[100] Salnikova L.E., Belopolskaya O.B., Zelinskaya N.I., Rubanovich A.V. (2013). The potential effect of gender in CYP1A1 and GSTM1 genotype-specific associations with pediatric brain tumor. Tumor Biol..

[101] Greenop K.R., Miller M., De Klerk N.H., Scott R.J., Attia J., Ashton L.J., Dalla-Pozza L., Bower C., Armstrong B.K., Milne E. (2014). Maternal Dietary Intake of Folate and Vitamins B6 and B12 During Pregnancy and Risk of Childhood Brain Tumors. Nutr. Cancer.

[102] Chiavarini M., Naldini G., Fabiani R. (2018). Maternal Folate Intake and Risk of Childhood Brain and Spinal Cord Tumors: A System-atic Review and Meta-Analysis. Neuroepidemiology.

[103] Bunin G.R., Kuijten R.R., Buckley J.D., Rorke L.B., Meadows A.T. (1993). Relation between Maternal Diet and Subsequent Primitive Neuroectodermal Brain Tumors in Young Children. N. Engl. J. Med..

[104] Sirikulchayanonta C., Madjupa K., Chongsuwat R., Pandii W. (2004). Do Thai women of child bearing age need pre-conceptional supplementation of dietary folate?. Asia Pac. J. Clin. Nutr..

[105] Sanghvi T.G., Harvey P.W., Wainwright E. (2010). Maternal iron-folic acid supplementation programs: Evidence of impact and imple-mentation. Food Nutr. Bull..

[106] Lucock M., Yates Z. (2005). Folic acid—Vitamin and panacea or genetic time bomb?. Nat. Rev. Genet..

[107] Kumawat R., Gowda S.H., Debnath E., Rashid S., Niwas R., Gupta Y., Upadaya A.D., Suri A., Chandra P.S., Gupta D.K. (2018). Association of Single Nucleotide Polymorphisms (SNPs) in Genes Encoding for Folate Metabolising Enzymes with Glioma and Meningioma in Indian Population. Asian Pac. J. Cancer Prev..

[108] Cartron P.-F., Hervouet E., Debien E., Olivier C., Pouliquen D., Menanteau J., Loussouarn D., Martin S.A., Campone M., Vallette F.M. (2012). Folate supplementation limits the tumourigenesis in rodent models of gliomagenesis. Eur. J. Cancer.

[109] Lubecka-Pietruszewska K., Kaufman-Szymczyk A., Stefanska B., Fabianowska-Majewska K. (2013). Folic acid enforces DNA methylation-mediated transcriptional silencing of PTEN, APC and RARbeta2 tumour suppressor genes in breast cancer. Biochem. Biophys. Res. Commun..

[110] Zgheib R., Battaglia-Hsu S.-F., Hergalant S., Quéré M., Alberto J.-M., Chéry C., Rouyer P., Gauchotte G., Guéant J.-L., Namour F. (2019). Folate can promote the methionine-dependent reprogramming of glioblastoma cells towards pluripotency. Cell Death Dis..

[111] Semmler A., Simon M., Moskau S., Linnebank M. (2006). The Methionine Synthase Polymorphism c.2756A>G Alters Susceptibility to Glioblastoma Multiforme. Cancer Epidemiol. Biomark. Prev..

[112] Pandith A.A., Qasim I., Zahoor W., Shah P., Bhat A.R. (2018). ACE I/D sequence variants but not MTHFR C677T, is strongly linked to malignant glioma risk and its variant DD genotype may act as a promising predictive biomarker for overall survival of glioma patients. Gene.

[113] Bethke L., Webb E., Murray A., Schoemaker M., Johansen C., Christensen H.C., Muir K., McKinney P., Hepworth S., Dimitropoulou P. (2007). Comprehensive analysis of the role of DNA repair gene polymorphisms on risk of glioma. Hum. Mol. Genet..

[114] Da Costa D.M., de Lima G.P.V., Faria M.H.G., Rabenhorst S.H.B. (2012). Polymorphisms of folate pathway enzymes (methylenetetrahydrofolate reductase and thymidylate synthase) and their relationship with thymidylate synthase expression in human astrocytic tumors. DNA Cell Biol..

[115] Kafadar A.M., Yilmaz H., Kafadar D., Ergen A., Zeybek U., Bozkurt N., Kuday C., Isbir T. (2006). C677T gene polymorphism of methylenetetrahydrofolate reductase (MTHFR) in meningiomas and high-grade gli-omas. Anticancer Res..

[116] Lu Q., Dai D., Zhao W., Wang L., Yue Z., Chen X., Han G., Hao B., Yang P., Deng A. (2013). Association between MTHFR 677C>T polymorphism and risk of gliomas: Evidence from a meta-analysis. Tumor Biol..

[117] Xu C., Yuan L., Tian H., Cao H., Chen S. (2013). Association of the MTHFR C677T polymorphism with primary brain tumor risk. Tumor Biol..

[118] Chen D., Dong J., Huang Y., Gao F., Yang X., Gong X., Lv X., Chu C., Wu Y., Zheng Y. (2017). Folate metabolism genetic polymorphisms and meningioma and glioma susceptibility in adults. Oncotarget.

[119] Zhang J., Zhou Y.-W., Shi H.-P., Wang Y.-Z., Li G.-L., Yu H.-T., Xie X.-Y. (2013). 5,10-Methylenetetrahydrofolate reductase (MTHFR), methionine synthase (MTRR), and methionine synthase reductase (MTR) gene polymorphisms and adult meningioma risk. J. Neuro-Oncol..

[120] Li R., Wang R., Li Y., Li X., Feng Y., Li Y., Jiang C. (2013). Association study on MTHFR polymorphisms and meningioma in northern China. Gene.

[121] Ding H., Liu W., Yu X., Wang L., Shao L., Yi W. (2014). Risk association of meningiomas with MTHFR C677T and GSTs polymorphisms: A meta-analysis. Int. J. Clin. Exp. Med..

[122] Han X.-Y., Wang W., Wang L.-L., Wang X.-R., Li G. (2017). Genetic variants and increased risk of meningioma: An updated meta-analysis. Onco.Targets Ther..

[123] Linnebank M., Semmler A., Moskau S., Smulders Y., Blom H., Simon M. (2008). The methylenetetrahydrofolate reductase (MTHFR) variant c.677C>T (A222V) influences overall survival of pa-tients with glioblastoma multiforme. Neuro Oncol..

[124] Cadieux B., Ching T.-T., Vandenberg S.R., Costello J.F. (2006). Genome-wide Hypomethylation in Human Glioblastomas Associated with Specific Copy Number Alteration, Methylenetetrahydrofolate Reductase Allele Status, and Increased Proliferation. Cancer Res..

[125] Faria G.M., Soares I.D.P., Salazar M.D., Amorim M.R., Pessoa B.L., Da Fonseca C.O., Quirico-Santos T. (2020). Intranasal perillyl alcohol therapy improves survival of patients with recurrent glioblastoma harboring mutant variant for MTHFR rs1801133 polymorphism. BMC Cancer.

[126] Bray F., Ferlay J., Soerjomataram I., Siegel R.L., Torre L.A., Jemal A. (2018). Global cancer statistics 2018: GLOBOCAN estimates of incidence and mortality worldwide for 36 cancers in 185 coun-tries. CA Cancer J. Clin..

[127] Nagini S. (2012). Carcinoma of the stomach: A review of epidemiology, pathogenesis, molecular genetics and chemoprevention. World J. Gastrointest. Oncol..

[128] Rawla P., Barsouk A. (2019). Epidemiology of gastric cancer: Global trends, risk factors and prevention. Gastroenterol. Rev..

[129] Karimi P., Islami F., Anandasabapathy S., Freedman N.D., Kamangar F. (2014). Gastric Cancer: Descriptive Epidemiology, Risk Factors, Screening, and Prevention. Cancer Epidemiol. Biomark. Prev..

[130] Cisło M., Filip A.A., Offerhaus G.J.A., Ciseł B., Rawicz-Pruszyński K., Skierucha M., Polkowski W.P. (2018). Distinct molecular subtypes of gastric cancer: From Laurén to molecular pathology. Oncotarget.

[131] Lauren P. (1965). The two histological main types of gastric carcinoma: Diffuse and so-called intes-tinal-type carcinoma. An at-tempt at a histo-clinical classification. Acta Pathol. Microbiol. Scand.

[132] Henson D.E., Dittus C., Younes M., Nguyen H., Albores-Saavedra J. (2004). Differential trends in the intestinal and diffuse types of gastric carcinoma in the United States, 1973–2000: Increase in the signet ring cell type. Arch. Pathol. Lab. Med..

[133] Network C.G.A.R. (2014). Comprehensive molecular characterization of gastric adenocarcinoma. Nature.

[134] Cristescu R., Lee J., Nebozhyn M., Kim K.-M., Ting J.C., Wong S.S., Liu J., Yue Y.G., Wang J., Yu K. (2015). Molecular analysis of gastric cancer identifies subtypes associated with distinct clinical outcomes. Nat. Med..

[135] Öksüz E., Görgişen G., Oto G., Özdemir H., Aras A., Öksüz M., Gülaçar İ.M., Demirkol M.H. (2020). Relationship between *MTHFR* Gene Polymorphisms and Gastrointestinal Tumors Development: Perspective from Eastern Part of Turkey. J. Invest. Surg..

[136] Han Z., Sheng H., Gao K., Fan Y., Xie X. (2021). Associations of the the *MTHFR* rs1801133 polymorphism with gastric cancer risk in the Chinese Han population. Biomed Rep..

[137] Gao C., Wu J., Ding J., Liu Y., Zang Y., Li S., Su P., Hu X., Xu T., Toshiro T. (2002). Polymorphisms of methylenetetrahydrofolate reductase C677T and the risk of stomach cancer. Zhonghua Liuxingbingxue Zazhi.

[138] Chen L., Lu N., Zhang B.-H., Weng L., Lu J. (2015). Association between the MTHFR C677T polymorphism and gastric cancer susceptibility: A meta-analysis of 5757 cases and 8501 controls. Oncol. Lett..

[139] Zintzaras E. (2006). Association of methylenetetrahydrofolate reductase (MTHFR) polymorphisms with genetic susceptibility to gastric cancer: A meta-analysis. J. Hum. Genet..

[140] Mazzuca F., Borro M., Botticelli A., Aimati L., Gentile G., Capalbo C., Maddalena C., Mazzotti E., Simmaco M., Marchetti P. (2015). Effect of MTHFR Polymorphisms on Gastrointestinal Cancer Risk in Italy. World J. Oncol..

[141] Dong L.M., Potter J.D., White E., Ulrich C.M., Cardon L.R., Peters U. (2008). Genetic susceptibility to cancer: The role of polymorphisms in candidate genes. JAMA.

[142] Lv L., Wang P., Sun B., Chen G. (2013). The polymorphism of methylenetetrahydrofolate reductase C677T but not A1298C contributes to gastric cancer. Tumor Biol..

[143] Dong X., Wu J., Liang P., Li J., Yuan L., Liu X. (2010). Methylenetetrahydrofolate Reductase C677T and A1298C Polymorphisms and Gastric Cancer: A Meta-analysis. Arch. Med. Res..

[144] Hosseini-Asl S.S., Pourfarzi F., Barzegar A., Mazani M., Farahmand N., Niasti E., Yazdanbod A., Didevar R., Akhavan H., Malekzadeh R. (2013). Decrease in gastric cancer susceptibility by MTHFR C677T polymorphism in Ardabil Province, Iran. Turk. J. Gastroenterol..

[145] Cui L.-H., Shin M.-H., Kweon S.-S., Kim H.N., Song H.-R., Piao J.-M., Choi J.-S., Shim H.J., Hwang J.E., Kim H.-R. (2010). Methylenetetrahydrofolate reductase C677T polymorphism in patients with gastric and colorectal cancer in a Korean population. BMC Cancer.

[146] Galván-Portillo M.V., Cantoral A., Oñate-Ocaña L.F., Chen J., Herrera-Goepfert R., Torres-Sanchez L., Hernandez-Ramirez R.U., Palma-Coca O., López-Carrillo L. (2009). Gastric cancer in relation to the intake of nutrients involved in one-carbon metabolism among MTHFR 677 TT carriers. Eur. J. Nutr..

[147] Tang C., Yu S., Jiang H., Li W., Xu X., Cheng X., Peng K., Chen E., Cui Y., Liu T. (2018). A Meta-Analysis: Methylenetetrahydrofolate Reductase C677T Polymorphism in Gastric Cancer Patients Treated with 5-Fu Based Chemotherapy Predicts Serious Hematologic Toxicity but Not Prognosis. J. Cancer.

[148] Wöhrer S.S., Raderer M., Hejna M. (2004). Palliative chemotherapy for advanced gastric cancer. Ann. Oncol..

[149] Zhao Y., Li X., Kong X. (2015). MTHFR C677T Polymorphism is Associated with Tumor Response to Preoperative Chemoradiotherapy: A Result Based on Previous Reports. Med. Sci. Monit..

[150] Meulendijks D., De Groot J.W.B., Los M., Boers J.E., Beerepoot L.V., Polee M.B., Beeker A., Portielje J.E., Goey S.H., De Jong R.S. (2016). Bevacizumab combined with docetaxel, oxaliplatin, and capecitabine, followed by maintenance with capecitabine and bevacizumab, as first-line treatment of patients with advanced HER2-negative gastric cancer: A multicenter phase 2 study. Cancer.

[151] Blank S., Rachakonda S., Keller G., Weichert W., Lordick F., Langer R., Springfeld C., Bruckner T., Becker K., Kumar R. (2014). A retrospective comparative exploratory study on two Methylentetrahydrofolate Reductase (MTHFR) polymorphisms in esophagogastric cancer: The A1298C MTHFR polymorphism is an independent prognostic factor only in neoadjuvantly treated gastric cancer patients. BMC Cancer.

[152] Kong S., Ye F., Dang Y., Hua Y., Zhang G. (2020). Association of MTHFR C677T polymorphism with severity and localization of chronic atrophic gastritis patients without Helicobacter pylori infection: A case control study. BMC Cancer.

[153] Saberi S., Zendehdel K., Jahangiri S., Talebkhan Y., Abdirad A., Mohajerani N., Bababeik M., Karami N., Esmaili M., Oghalaie A. (2012). Impact of Methylenetetrahydrofolate Reductase C677T Polymorphism on the Risk of Gastric Cancer and Its Interaction with Helicobacter pylori Infection. Iran. Biomed. J..

[154] Wu X., Peng B., Qian K., Zhang W., Min J., Zhang M., Zeng F., Wang Z. (2021). The combination of methylenehydrofolate reductase C677T polymorphism screening and gastrointestinal tumor markers detection may be an early screening method for gastrointestinal cancer related to helicobacter pylori infection. Genes Dis..

[155] Kakkoura M.G., Sokratous K., Demetriou C.A., Loizidou M.A., Loucaides G., Kakouri E., Hadjisavvas A., Kyriacou K. (2017). Mediterranean diet-gene interactions: A targeted metabolomics study in Greek-Cypriot women. Mol. Nutr. Food Res..

[156] Shiao S.P.K., Grayson J., Yu C.H., Wasek B., Bottiglieri T. (2018). Gene Environment Interactions and Predictors of Colorectal Cancer in Family-Based, Multi-Ethnic Groups. J. Pers. Med..

